# Development of a self-administered early inflammatory arthritis detection tool

**DOI:** 10.1186/1471-2474-11-50

**Published:** 2010-03-17

**Authors:** Mary J Bell, Ruben Tavares, Francis Guillemin, Vivian P Bykerk, Peter Tugwell, George A Wells

**Affiliations:** 1 Division of Rheumatology, Department of Medicine, University of Toronto, Sunnybrook Health Sciences Centre, M1-401 2075 Bayview Avenue, Toronto, M4N 3W5, Canada; 2 Medical Sciences - Physiology and Pharmacology, McMaster University, 612-25 Charlton Avenue East, Hamilton, L8N 1Y2, Canada; 3 Centre d'Epidemiologie Clinique, Centre Hospitalier Universitaire de Nancy, 92 Avenue du Maréchal de Lattre de Tassigny, Nancy, 54035 CEDEX France; 4 Division of Rheumatology, Department of Medicine, University of Ottawa, Centre of Global Health, 1 Stewart Street, Ottawa, K1N 6N5, Canada; 5 Department of Epidemiology and Community Medicine, University of Ottawa, H1-1 40 Ruskin Street, Ottawa, K1Y 4W7, Canada

## Abstract

**Background:**

Barriers to care limit the potential benefits of pharmacological intervention for inflammatory arthritis. A self-administered questionnaire for early inflammatory arthritis (EIA) detection may complement contemporary triage interventions to further reduce delays to rheumatologic care. The objective of this study was to develop a self-administered EIA detection tool for implementation in pre-primary care settings.

**Methods:**

A core set of dimensions and constructs for EIA detection were systematically derived from the literature and augmented by investigative team arbitration. Identified constructs were formulated into lay language questions suitable for self-administration. A three-round Delphi consensus panel of EIA experts and stakeholders evaluated the relevance of each question to EIA detection and suggested additional items. Questions accepted by less than 70% of respondents in rounds one or two were eliminated. In round three, questions accepted by at least 80% of the panel were selected for the tool.

**Results:**

Of 584 citations identified, data were extracted from 47 eligible articles. Upon arbitration of the literature synthesis, 30 constructs encompassing 13 dimensions were formulated into lay language questions and posed to the Delphi panel. A total of 181 EIA experts and stakeholders participated on the Delphi panel: round one, 60; round two, 59; and, round three, 169; 48 participated in all three rounds. The panel evaluated the 30 questions derived from the literature synthesis, suggested five additional items, and eliminated a total of 24. The eleven-question instrument developed captured dimensions of articular pain, swelling, and stiffness, distribution of joint involvement, function, and diagnostic and family history.

**Conclusions:**

An eleven-question, EIA detection tool suitable for self-administration was developed to screen subjects with six to 52 weeks of musculoskeletal complaints. Psychometric and performance property testing of the tool is ongoing.

## Background

Barriers to care [1-5] continue to suppress the therapeutic advantages of early pharmacological intervention in inflammatory arthritis (IA) with disease-modifying anti-rheumatic drugs (DMARDs) [6-8]. Pronounced barriers exist along the entire care pathway. Prior to primary care, patient preferences, psychosocial issues, and interrelationship issues with primary care practitioners (PCPs) may negatively impact health seeking behaviour [9-11]. In primary care, PCPs have the formidable task of detecting an IA incidence of 0.05 to 0.2% in the absence of sensitive laboratory and diagnostic imaging tests [12,13]. Acute symptomatic response to non-steroidal anti-inflammatory drugs (NSAIDs) and disease presentation in undifferentiated or spontaneously remitting forms may further delay diagnosis, referral and treatment [14,15]. In addition, PCPs may have insufficient musculoskeletal (MSK) training in residency or continuing medical education (CME) opportunities to effectively detect and manage IA [15-20]. Although rheumatology referral may be hampered by shortages of rheumatologists and long referral waiting lists in some regions [9-11], the majority of the delays to DMARD treatment occur prior to referral [1].

Several approaches have been developed to minimize these barriers. These include public awareness programs, CME programs to improve MSK clinical management in primary care [21,22], early referral guidelines [14,23-25], and EIA triage tools [26-29]. A synergistic intervention utilizing patient self-detection has been developed in the current study.

The objective of this study was to develop a self-administered EIA detection tool encompassing dimensions of stage-one case ascertainment to accelerate access to appropriate care for IA. The two stages of case ascertainment include 1) detection of suspected cases, and 2) confirmation of the clinical diagnosis [30]. Clinical examinations, comprised of history-taking and physical examination, are used in stage-one. Laboratory and diagnostic imaging investigations in EIA are limited to stage-two due to their high cost and low sensitivity. As a stage-one case ascertainment intervention, the *a priori *criteria for the EIA detection tool were 1) inclusion of clinical history and physical examination elements suitable for self-administration, 2) exclusion of items requiring medical intervention to enable pre-primary care self-assessment, and 3) simplicity and brevity, to render the tool applicable to a broad demographic.

## Methods

### Literature Search

**Figure 1:**
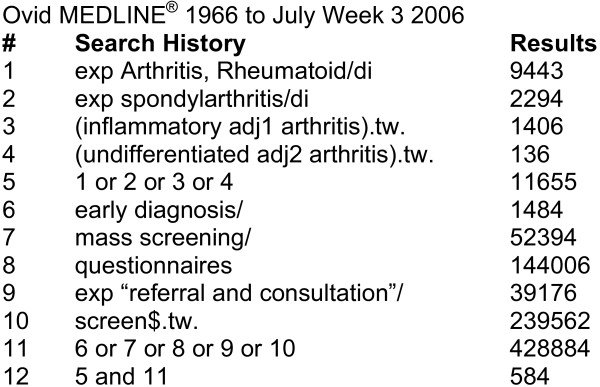
**Literature search strategy to identify stage-one case ascertainment constructs of early inflammatory arthritis**.

A structured literature search was conducted to derive dimensions and constructs relevant to EIA detection. The National Library of Medicine citation index, MEDLINE (1966 to July Week 3, 2006), was searched using a combination of medical subject headings and keywords: ["exp Arthritis, Rheumatoid/di" or "exp Spondylarthritis/di" or "(inflammatory adj1 arthritis).tw." or "(undifferentiated adj2 arthritis).tw."] and ["early diagnosis" or "mass screening" or "exp 'referral and consultation" or "screen$.tw."] (Figure 1). A Cochrane Collaboration MSK Group reference librarian developed and conducted the search strategy. The criteria for article selection included the investigation of prognostic indicators of EIA, or questionnaires developed for early or established IA detection. Articles in languages other than English, French or German were excluded.

Independently, two rheumatologists sequentially reviewed the citation title, abstract, and full article. At each stage of the review, where sufficient information was available to determine the ineligibility of articles, they were excluded. After the independent selection of eligible articles, discrepancies were resolved by consensus between the two reviewers. A third-party arbitrator was selected to settle non-consensus items but did not need to be used. Data extraction was conducted independently by the two reviewers and consensus was used to resolve discrepancies. The consensus list of dimensions and constructs for EIA detection were extracted and adjudicated by the investigative team.

The investigative team met to evaluate the relevance of the identified items to the objective, target population, and proposed mode of administration of the tool. The literature synthesis was supplemented with additional dimensions and constructs derived from clinical experience, guidelines for MSK examination [31,32], and classification criteria for IA [33-35]. The investigative team arbitrated on the items identified to select dimensions and constructs of stage-one case ascertainment and to render the tool suitable to self-administration in pre-primary care settings. Selected items were formulated into grade eight reading level questions using the Flesch-Kincaid Grade level in Microsoft Word 2003 (Redmond, WA). Where available, questions from pre-existing IA tools were adapted for self-administration in the current tool [30].

### Delphi Consensus Panel

Early inflammatory arthritis health professionals (EIA experts) were solicited for participation in a three-round Delphi consensus panel to evaluate the relevance of the derived questions for EIA detection. Experts were identified from the literature search, abstracts from the annual meetings of the ACR, Canadian Rheumatology Association (CRA), and EULAR, and through nomination by participant EIA experts. Members of the ACR, American College of Family Physicians (AAFP), American College of Physicians (ACP), Association of Rheumatology Health Professionals (ARHP), Outcome Measures in Rheumatology (OMERACT), patient advocacy groups, and United States Bone and Joint Decade (USBJD) were solicited for participation as additional stakeholders. Delphi panel participants evaluated the relevance of each question to EIA detection ("yes"/"no"), and suggested additional items.

Thresholds for question acceptance by the Delphi panel were set *a priori*: round one, ≥ 0.70; round 2, ≥ 0.70; and, round 3, ≥ 0.80. Percent acceptance was used to assess the true relevance of questions to EIA detection. In round one, the lower threshold excluded the least frequently accepted questions. In round two, a selection of questions below the threshold in round one was retested to determine the inter-round consistency of the panel. New questions derived from round one feedback were also tested. In round three, questions above the threshold from rounds one or two were re-evaluated by the panel. In round three, a higher threshold was used to minimize the total number of questions while selecting those of greatest overall relevance.

The primary analysis population included all Delphi panel participants. The secondary analysis population included participants of all three rounds of the Delphi panel. Differences in the acceptance of questions between the two analysis populations were arbitrated by the investigative team to derive the EIA detection tool.

## Results

**Figure 2:**
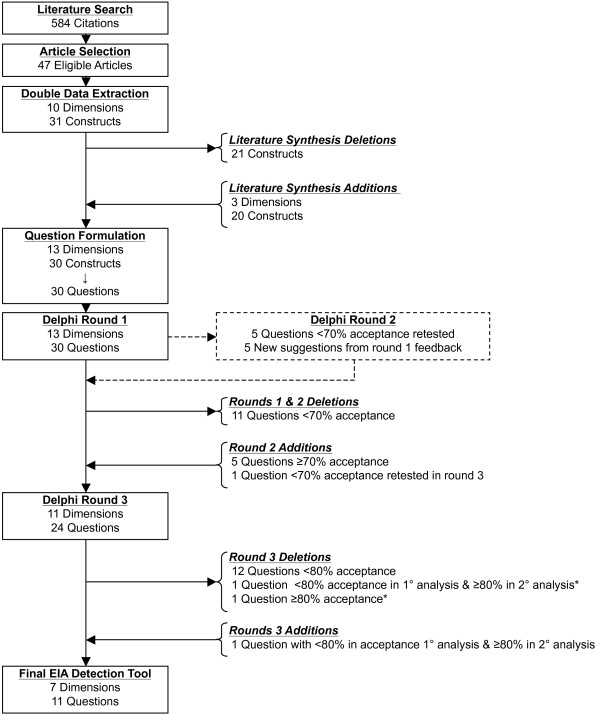
**Study flow diagram**. The diagram illustrates the flow of the dimensions, constructs, and derivative questions from a synthesis of the literature, revisions based on investigative team arbitration, and three-round Delphi consensus panel acceptance. EIA = early inflammatory arthritis. *Combined into eligibility criteria for tool administration

The processes of literature synthesis, investigative team arbitration, and Delphi panel acceptance resulted in the development of eleven EIA stage-one case ascertainment questions (Figure 2).

### Literature Synthesis

A total of 584 citations were identified from the literature search strategy. Upon independent review by the two reviewers and consensus on discrepant selections, 47 articles were selected for the identification of dimensions and constructs for EIA detection. Ten unique dimensions were derived from the literature synthesis: demographics; pain; swelling; stiffness; fatigue; nodules; function; laboratory and diagnostic imaging; genetics; and, diagnostic history (including constitutional symptoms). Thirty-one constructs were identified and categorized under the derived dimensions. Of these, eleven specific laboratory and diagnostic imaging tests were determined to be inappropriate for self-administration in pre-primary care settings. In place of these, items regarding a history of having had a "blood test for RA" and "x-rays of your hands or wrists" were proposed by the investigative team. Likewise, although physical function was evaluated as relevant to the detection of EIA, specific, extensive instruments (e.g. HAQ) were inappropriate for a brief self-administered instrument. To address the dimension of function, a question pertaining to activities of daily life was proposed. Two specific genetic factors (HLA B27; HLA DRB1) were replaced with items pertaining to family history of IA. Psychosocial and socioeconomic dimensions were arbitrated to be access to care issues and not dimensions of EIA detection.

### Investigative Team Arbitration of Literature Synthesis

Utilizing clinical experience, MSK guidelines, IA classification criteria, and pre-existing IA questionnaires, the investigative team proposed the following additional dimensions as relevant to EIA detection: treatment history; characterization of symptom onset; and, distribution of joint involvement. Additional demographic constructs included age, recent pregnancy, and recent weight loss. In total, the investigative team differentiated 13 dimensions of EIA detection, which were captured by 30 specific constructs. The 30 constructs were formulated into individual, grade eight reading level questions in second-person narrative. Over the subsequent three-round Delphi consensus panel, 35 questions encompassing 13 dimensions were evaluated for their relevance to EIA detection (Table 1).

**Table 1:** Delphi panel acceptance of questions for an early inflammatory arthritis detection tool

Elements and Questions	Delphi Round*			EIA Tool (X)
		
	1 (n = 60)	2 (n = 59)	3 (n = 169)	
Demographics				
1. In which month and year were you born?	66.7	-	-	-^†^
2. What is your gender, male or female?	75.0	-	54.4	-^†^
3. If female, have you been pregnant or given birth within the last year?	63.3	-	-	-
4. Have you smoked or have you been exposed to smoke on a regular basis in your life?^‡^	55.0	54.2	26.6	-
5. Over the past year, have you had more than 10 lbs (5 kg) weight loss without trying?	-	57.6	-	-
Articular Pain				
6. Do you have pain in your joints?	91.7	-	91.1	X
7. Do you have pain in your wrists and hands?	95.0	-	92.3	X
8. Do you have pain in the ball of your foot?	71.7	-	65.7	-
9. Do you have pain in your neck, your back, your buttocks or the muscles in your legs?	63.3	-	-	-
Articular Swelling				
10. Are your hands or wrists swollen?	98.3	-	91.1	X
11. Are your rings still fitting?^‡^	56.7	44.1	-	-
12. Do you have trouble with your shoes fitting?	43.3	20.3	-	-
Articular Stiffness				
13. Do you have trouble making a fist?	93.3	-	85.2	X
14. Are your joints stiff in the morning?	96.7	-	95.3	X
15. Do you have a feeling of back stiffness in the morning?^‡^	61.7	74.6	78.1	-
16. From the time you wake in the morning, how many minutes does it take for your joints to move more freely, less than 30 minutes or more than 30 minutes?^‡^	95.0	-	91.7	X
Distribution of Joint Involvement				
17. At any time have your lower limbs been affected, such as your groin knees, ankles, or feet?	63.3	-	-	-
18. Are the same joints involved on both sides of your body?	-	88.1	87.6	X
Characterization of symptom onset				
19. For how long have you had these symptoms, less than 1 year or more than 1 year?^‡^	86.7	-	79.3^§^	-**
20. Did your bone and joint problem come on suddenly?^‡^	-	76.3	80.5	-**
Nodules				
21. Have you developed any new lumps or bumps on your arms or legs?	66.7	-	-	-
Function				
22. Have important activities in your life been affected because of bone or joint problems, such as having difficulty with personal care or having to make a change regarding leisure or work activities?	80.0	-	87.6	X
Fatigue				
23. Do you find that you are getting tired earlier in the day than you used to?	68.3	-	-	-
Laboratory Test and Diagnostic Imaging History				
24. Have you had a blood test for rheumatoid arthritis?	75.0	-	63.3	-
25. Have you had x-rays of your hands or wrists?	68.3	-	-	-
Diagnostic History				
26. Have you ever been told that you have rheumatoid arthritis?	88.3	-	72.8^§^	X
27. Have you seen an arthritis specialist or rheumatologist in the past year?	80.0	-	71.6	-
28. Have you been diagnosed with a rash called psoriasis?	90.0	-	81.7	X
29. Have you had a recent viral or other infection or illness?^‡^	-	76.3	76.9	-
Family History				
30. Does anyone in your family have rheumatoid arthritis?	91.7	-	93.5	X
31. Does anyone in your family have a rash called psoriasis?	78.3	-	79.9	-
Treatment History				
32. Have you used anti-inflammatory drugs to manage your arthritis?	80.0	-	79.3	-
33. Have you used disease-modifying anti-rheumatic drugs (DMARDS) to manage your arthritis?	71.7	-	65.7	-
34. Have you used non-pharmacological (non-drug) methods to manage your arthritis?	61.7	54.2	-	-
35. Do you take pills on a daily basis to make your pain or stiffness feel better?	-	79.7	72.8	-

*In rounds 1 and 2, a cut-off of <70% was used to eliminate questions from the EIA detection tool. In round 3, a cut-off of <80% was used.

^†^Age and gender are captured as demographic assessments on the EIA detection tool.

^‡^Phrasing of question changed in round two or three.

^§^85.4% Acceptance by respondents involved in all three rounds of the Delphi panel (secondary analysis population).

**Symptom duration items were modified into eligibility criteria for tool administration. Persons with musculoskeletal complaints for a duration of at least six weeks but no more than 52 weeks are eligible to self-administer the tool.

### Delphi Panel: Participants

**Figure 3:**
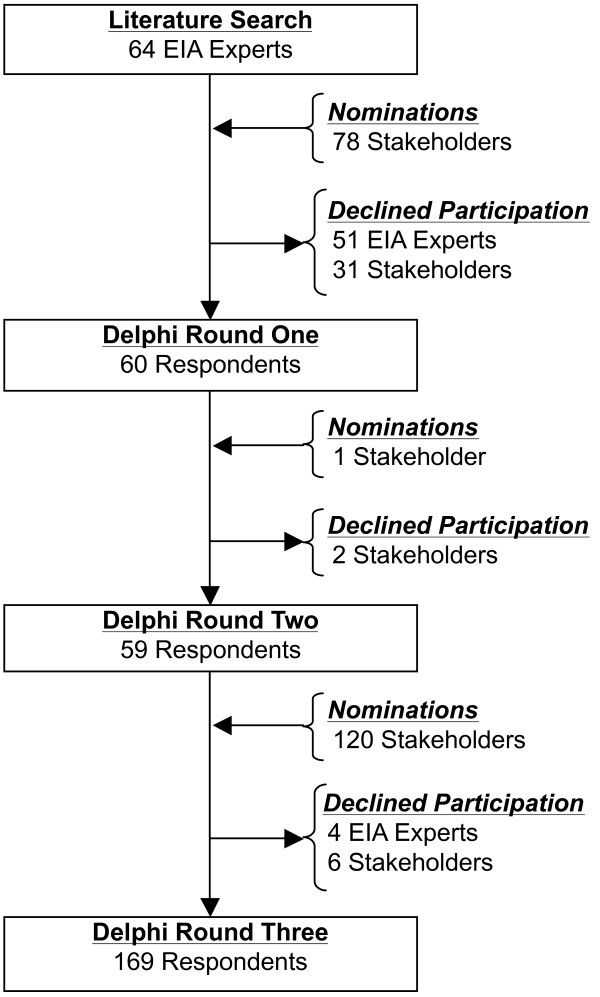
**Delphi consensus panel participant flow diagram**. EIA = early inflammatory arthritis

A total of 181 EIA experts and stakeholders participated in the Delphi panel (Figure 3). Thirteen of 64 solicited EIA experts participated in the Delphi panel and nominated 13 additional participants. Five of the 13 nominations participated on the Delphi panel. The investigative team identified an additional 65 representatives from stakeholder groups, 42 of which participated in the Delphi panel. Over the course of the three-round Delphi panel, an additional 121 stakeholders participated. Overall, nine EIA experts and 39 stakeholders participated in all three Delphi panels. Delphi panel participants represented a broad demographic of countries and health care disciplines (Table 2).

**Table 2:** Delphi consensus panel participant characteristics

Characteristics	Overall (n = 181)	Participants in all 3 Rounds (n = 48)	Additional Participants (n = 133)
Gender			
Female	59.1	52.1	63.6
Male	40.9	47.9	36.4
Country/Region of practice			
United States of America	37.0	8.3	9.1
Canada	35.4	8.3	2.5
Romania	7.7	6.3	9.1
Austria	6.1	0.0	6.6
United Kingdom	4.4	4.2	4.1
Australia	3.9	4.2	4.1
Turkey	2.2	2.1	2.5
Spain	1.7	2.1	1.7
Switzerland	1.7	2.1	1.7
Primary discipline			
Rheumatologist	38.1	31.2	40.6
Physical/Occupational Therapy	19.9	18.8	20.3
Patient Advocate	13.8	2.1	18.0
Other Physician*	12.7	14.6	12.0
Physician Assistant/Nurse Practitioner	12.2	25.0	7.5
Rheumatology Nurse	3.3	8.3	1.5
Career Stage^†^			
Mid career	67.9	63.8	71.1
Late career	32.1	36.2	27.8
Urban or Rural^†^			
Urban	85.3	87.2	84.5
Rural	14.7	12.8	14.4
Majority of Time Spent^†^			
Clinical	67.9	59.6	74.2
Academic	32.1	40.4	25.8
Primary Work Setting^†^			
Institutional	73.7	68.1	76.3
Private Practice	26.3	31.9	23.7

*Family medicine, physical medicine and rehabilitation, internal medicine and paediatrics

^†^Characteristics not applicable to patient advocates.

### Delphi Panel: Round one

Thirty questions were posed to the 60 Delphi panel round one participants. Eighteen questions met the 70% threshold. Five new items were suggested by round one participants that represented unique constructs relating to back stiffness, characterization of symptom onset, distribution of joint involvement, infection, and use of medications. Five new questions were developed by the investigative team and introduced in round two to evaluate the relevance of these suggestions.

### Delphi Panel: Round two

In Round two, ten questions were posed to the panel. The round two panel was comprised of round one participants and one additional nominated participant. Two members of the round one panel did not respond in round two. A selection of five of the 12 questions below the round one threshold and the five new questions derived from round one suggestions were tested in round two. Five of the ten questions tested met the round two threshold (four new suggestions and one rephrased question from round one). Through rounds one and two, 23 questions met the 70% threshold and were re-tested in round three. An additional question below the round one and two threshold was rephrased and tested in round three.

### Delphi Panel: Round three

A total of 24 questions were evaluated by 169 participants in the Delphi panel round three. Eleven questions met the round three threshold as evaluated by all round three participants. All questions above the threshold in primary analysis population remained above the threshold in the secondary analysis population. Two additional questions (Table 1, no. 20 and no. 24) met the round three threshold in the secondary analysis population only. Question no. 20 was combined with no. 21 and introduced as eligibility criteria for tool administration. The investigative team arbitrated to keep questions no. 24 in the tool.

### EIA Detection Tool

**Figure 4:**
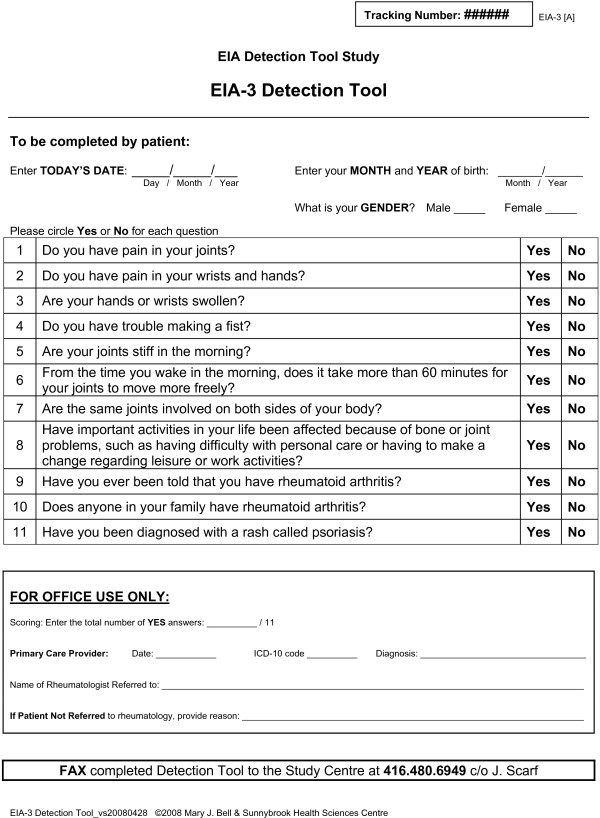
**Early inflammatory arthritis detection tool**.

The tool included eleven questions encompassing seven dimensions of EIA detection and captured demographic characteristics of the target population (Figure 4). The dimensions captured included articular pain, swelling and stiffness, distribution of joint involvement, function, and diagnostic and family history of IA. Two questions pertained to pain: one general, "Do you have pain in your joints?"; and one joint-specific, "Do you have pain in your wrists and hands?". One question pertained to swelling: "Are your hands or wrists swollen?". Stiffness was captured by three questions: one general, "Do you have trouble making a fist?"; one pertained to time of onset, "Are your joints stiff in the morning?"; and one included duration within the construct, "From the time you wake in the morning, does it take more than 60 minutes for your joints to move more freely?". One question pertained to the distribution of joint involvement: "Are the same joints involved on both sides of your body?". Physical function was captured by one question: "Have important activities in your life been affected because of bone or joint problems, such as having difficulty with personal care or having to make a change regarding leisure or work activities?". A question regarding primary care diagnostic history was included: "Have you ever been told you have rheumatoid arthritis?". Family history was captured by two questions: "Does anyone in your family have rheumatoid arthritis?"; and, "Have you been diagnosed with a rash called psoriasis?". The tool also included two eligibility criteria restricting the detection population to persons with at least six weeks and less than 52 weeks of musculoskeletal symptoms.

## Discussion

A self-assessment instrument was developed to promote primary care health-seeking behaviour and accelerate the referral of incident cases of IA to rheumatology. The eleven-question tool captures the dimensions of articular pain, swelling, and stiffness, distribution of joint involvement, function, and diagnostic and family history. It was designed for self-administration by persons with six to 52 weeks of MSK complaints. The focus of the current study was the development of a tool with content and construct validity. Studies to investigate a scoring algorithm and the tool's performance and psychometric properties are ongoing.

The content validity of the EIA detection tool is assessed in the context of stage-one case ascertainment. The EIA detection tool directly captures seven of 13 dimensions relevant to EIA detection. Dimensions were derived by the investigative team to organize data from the literature synthesis. Constructs were defined as elements of prognostic factors and clinical measures requiring formulation into self-assessment questions. The content of the tool is consistent with the dimensions of clinical examination as described in guidelines for the assessment of new-onset MSK complaints [31,32] and classification criteria for IA [33-35]. Although there is paucity in the predictive validity of individual measures of clinical examination [32], these are clinically relevant measures applicable to stage-one case ascertainment.

The construct validity of the tool is qualitatively ascribed by comparisons to IA clinical examination measures found in classification criteria, early referral recommendations, prevalence questionnaires, and triage tools. Two prominent IA classification criteria include the 1987 ACR criteria for RA [33] and the European Spondylarthopathy Study Group (ESSG) criteria for SpA [34]. Although the value of these criteria in the detection of EIA is questionable [36-39], new criteria directed at early rheumatoid arthritis detection have been proposed by an ACR/EULAR collaboration and include joint involvement, symptom duration and serological markers in its scoring algorithm [40]. Physical examination constructs from these and other IA criteria [35] appear in the tool. Questions pertaining to the 1987 ACR criteria constructs of morning stiffness, joint areas involved, swelling, and symmetry appear in the EIA detection tool and dimensions of joint pain, stiffness, swelling, and psoriasis are applicable to the ESSG criteria. Through the Delphi panel, specific laboratory and diagnostic imaging tests, and items pertaining to nodules, buttock pain, and constitutional symptoms were eliminated.

The dimensions and constructs in the tool are also similar to those captured in prominent early referral to rheumatology recommendations for IA. The early referral recommendation for RA includes at least 30 minutes duration of morning stiffness, at least three swollen joints, or tenderness of either the metacarpophalangeal or metatarsophalangeal joints [24]. The early referral recommendation for ankylosing spondylitis includes inflammatory back pain, genetic predisposition (HLA B27), and sacroiliitis [41,42]. With exception to the laboratory and diagnostic imaging tests applicable to stage-two case ascertainment, the dimensions of pain, swelling, and stiffness in the EIA detection tool encompass the clinical examination dimensions in early referral recommendations for IA conditions. Similarly, there is some overlap between the EIA detection tool and clinical examination dimensions found in existing interviewer-administered IA prevalence questionnaires and triage tools [26-29]. Few tools, classification criteria, or early referral recommendations include health assessment instruments, such as the HAQ or multi-dimensional HAQ (MDHAQ). To reduce questionnaire burden on the population, such tools may be more suitable for stage-two case ascertainment as proposed by Emery [43]. Cumulatively, overlaps between the content of the EIA detection tool and measures of classifying, recommending referral of, or triaging IA conditions, support the construct validity of the tool.

The EIA detection tool encompasses many unique features. Foremost, the tool targets the pre-primary care population, whereas triage tools focus on prioritizing existing referrals [26-29]. Although synergistic to triage instruments, the EIA detection tool offers the potential advantage of detecting cases that delay or avoid primary care, or are otherwise not considered for rheumatologic care. Among IA instruments, the mode of self-administration is also unique. The questions have been formulated in lay language, kept brief, and have unidirectional, dichotomous "yes"/"no" response options. The simplicity and brevity of the tool are expected to facilitate its adoption in pre-primary care settings.

The population for the EIA detection tool is restricted to persons with six to 52 weeks of MSK complaints. As suggested by preliminary performance testing, this restriction increases the pre-test probability of IA from the incidence of 0.05-to-0.2% to 25% (data not presented). Hypothetically, if the general population was screened with a tool with 99% sensitivity and 99% specificity the positive predictive value (PPV) would range from ten to 30% [44]. With a pre-test probability of EIA of 25%, a tool with 99% sensitivity and specificity results in a PPV for EIA that exceeds 95% [44]. Restricting the population augments the pre-test probability of EIA cases to a high percentage that improves the performance properties of the tool.

Others have applied restrictions as well. The early referral recommendation for ankylosing spondylitis is restricted to patients presenting with more than three months of low back pain and symptom onset prior to 45 years of age [41,42]. Age-based restrictions for breast cancer, osteoporosis, and other diseases are common and accepted as well. Despite the acceptable use of restrictions, the tool may be modified for detecting EIA in an unrestricted population. The symptom duration requirements currently included as restrictions may by re-introduced as questions, i.e. "Have you had [joint or back pain, swelling or stiffness] for more than six weeks?" and "Have you had [joint or back pain, swelling or stiffness] for less than one year?". Only respondents answering "yes" to both conditional questions, would be further evaluated for EIA. This strategy would leverage the advantages of pre-test probability augmentation and simplify field implementation.

Some unique dimensions were not selected for the tool due to not meeting the Delphi panel threshold. Although symptomatic response to NSAIDs has been reported as a prognostic indicator of IA [14,35], the investigative team refrained from overruling the Delphi panel failure to accept the NSAID question to avoid the indirect endorsement of ineffective IA treatments. There was insufficient evidence in the literature for the validity of fatigue as a dichotomous predictive measure of EIA to overturn the Delphi panel's failure to accept the item. In addition, laboratory and diagnostic imaging tests important in stage-two were excluded owing to the central aim of developing a stage-one case ascertainment instrument.

The performance properties of this tool are outstanding. Studies to evaluate the performance and psychometric properties of the EIA detection tool are underway. The intra-rater reliability, internal consistency, and discriminant validity are currently being tested. A scoring algorithm to optimize the sensitivity, specificity, and positive and negative predictive values of the tool is also being investigated. Ultimately, an evaluation of the tool's psychometric, and performance properties, is required to support the content and construct validity reported in the current study.

## Conclusions

A self-administered tool with content and construct validity has been developed for stage-one case ascertainment of EIA. The tool includes eleven brief, simple language questions evaluated as relevant by a high percentage of EIA experts and stakeholders. Its self-assessment design allows it to be implemented pre-primary care, where the greatest impact of reducing delays to care is expected.

## Competing interests

Grant support from the American College of Rheumatology Quality Measure Committee. The authors have no competing interests to disclose.

## Authors' contributions

MB, FG, VPB, PT and GW were involved with study design, data acquisition, analysis and interpretation, and manuscript preparation. RT was involved with analysis and interpretation, and manuscript preparation. All authors read and approved the final manuscript.

## Pre-publication history

The pre-publication history for this paper can be accessed here:

http://www.biomedcentral.com/1471-2474/11/50/prepub
